# Fluid Pressure Sensing Strategy Suitable for Swallowing Soft Gripper

**DOI:** 10.3390/s26030960

**Published:** 2026-02-02

**Authors:** Mingge Li, Wenxi Zhang, Quan Liu, Zhongjun Yin

**Affiliations:** The School of Mechanical Engineering, University of Science and Technology Beijing, Beijing 100083, China; lmgpigeon@163.com (M.L.); wenxi_zjj@163.com (W.Z.); hbnu_liuquan@163.com (Q.L.)

**Keywords:** soft sensors, soft gripper, fluid pressure, feedback

## Abstract

Soft grippers exhibit excellent adaptability in handling objects of various shapes. However, due to the large deformation and high compliance of their constituent materials, the integration of sensing capabilities has long been a major research challenge. Based on the swallowing-type soft gripper proposed in previous work, this study explores the gripper’s capability to perceive object information by leveraging the characteristic that the sealed cavity undergoes volume change due to compression by the object during swallowing, thereby altering the pressure of the internal fluid medium. By establishing the geometric configuration of the sealed cavity composed of elastic membranes, the volume-pressure variation sensing model during the object swallowing process was derived. The performance of this sensing method was tested, and the application of the fluid pressure sensing strategy in closed-loop control was demonstrated, including the classification of objects by shape and sorting by size. This work provides a solution for the object shape-adaptive swallowing-type soft gripper to achieve sensory grasping functionality.

## 1. Introduction

Soft grippers represent a crucial research direction in the field of soft robotics [1,2]. Compared with rigid grippers, soft grippers are fabricated from highly compliant materials, endowing them with exceptional flexibility and adaptability. Various types of soft grippers with distinct configurations have been developed, such as soft fingers mimicking the motion of human fingers [3,4,5], wrapping grippers inspired by elephant trunks or plants [6,7,8], and suction-based grippers emulating octopus suckers [9,10]. However, these soft grippers often require precise grasping positions during object manipulation or impose high surface smoothness requirements on the objects. Therefore, general-purpose grippers capable of automatically adapting to the shape of objects exhibit superior performance when handling objects of unknown shapes and sizes.

Biological swallowing behavior provides a reliable reference for addressing this issue. When organisms ingest food, they utilize the large deformability of the oral cavity or intestines to swallow food of various shapes through peristaltic motion. Accordingly, a series of swallowing-peristaltic soft grippers has been proposed. Zhu et al. [11] fixed a pull rope within the elastic membrane of a general-purpose gripper; pulling the rope forms a concave vacuum cavity in the gripper. Inspired by the feeding behavior of sea anemones, Zang et al. [12] designed a swallowing-type soft gripper composed of a double-layer cylindrical tube, where the inverted membrane swallows the object into its interior, driven by frictional force. Inspired by the food-capturing mechanism of bloodworms, Sui et al. [13] developed a fluid-filled swallowing gripper. Drawing on earthworms’ food-capturing method, Li et al. [14] proposed a swallowing-type soft gripper of different configurations, where the elastic membrane inverts inward and outward, driven by a central rod to swallow the object. Li et al. [15] filled the elastic membrane with a particle suspension, which enables particle jamming after swallowing, thereby enhancing the gripper’s load-bearing capacity. Although these improvements have addressed the challenges of general-purpose grippers in handling objects of unknown shapes—such as issues related to grasping accuracy and reliability—the lack of sensing capabilities prevents them from performing high-level manipulation tasks, such as object shape recognition and object classification.

There are numerous sensing methods applicable to soft grippers, such as the resistive-voltage type [16], capacitive type [17], optical type [18], and magnetic field type [19]. However, during the object-grasping process of swallowing grippers, their elastic membranes undergo large deformation and reciprocating motion. Embedding sensors such as strain gauges or optical fibers into the elastic membrane will inevitably affect its own motion, and the large-deformation motion mode will significantly reduce the sensor’s lifespan. The sensing method that realizes the perception of the external environment or load by utilizing the fluid pressure change in the sealed cavity is referred to as pressure sensing. Zou et al. [20] modified existing fluid-driven actuators and proposed a universal sensing strategy, which achieves perception by measuring the fluid input required to activate the actuator for devices interacting with the environment. Barvenik et al. [21] developed a hemispherical membrane-based general-purpose gripper that judges the size of the grasped object by detecting the pressure change generated when the membrane contacts the object. Joshi et al. [22] measured the pressure of pneumatic actuators under different bending degrees and demonstrated a bijective relationship between the properties of soft actuators and the outputs of pressure, volume, displacement, and force. Block et al. [23] extended pressure sensing to human–robot interaction scenarios, installing inflatable air cushions on the front and rear of rigid robots; the pressure response changes generated by actions such as hugging humans serve as the basis for robot reaction judgment. Truby et al. [24] designed a sensor composed of nine air-filled flexible tubes, which can detect deformation information such as stretching, bending, and torsion. Tawk et al. [25] arranged pneumatic tactile sensing chambers (pTSCs) on the inner side of rope-driven soft fingers, enabling real-time detection of finger bending states and perception of information such as the surface texture and weight of touched objects. Furthermore, various shapes of pneumatic sensing chambers have been developed, capable of responding to four main mechanical input modes: compression, bending, torsion, and expansion. Wang et al. [26] proposed a method utilizing symmetrically arranged bellows structures: actively controlling one bellows air chamber to extend or contract, thereby prompting the other equidistant bellows to contract or extend, to realize active and passive mechanical perception of the manipulator. Gong et al. [27] fabricated a pneumatic tactile sensor prototype that can perceive the softness and surface roughness of objects in tactile applications.

However, such sensing methods based on fluid pressure are mostly limited to finger-type soft grippers, and pressure sensing methods for swallowing-wrapping type soft grippers have not been reported. In this work, by leveraging the characteristic that during the object grasping process of the swallowing-type soft gripper, the elastic membrane of the sealed cavity deforms due to swallowing the object, which alters the volume of the sealed cavity and thereby induces variations in fluid pressure, the information of the swallowed object can be determined by detecting the trend of such variations.

## 2. Design and Method

### 2.1. Principle Prototype of the Swallowing Gripper

**Figure 1:**
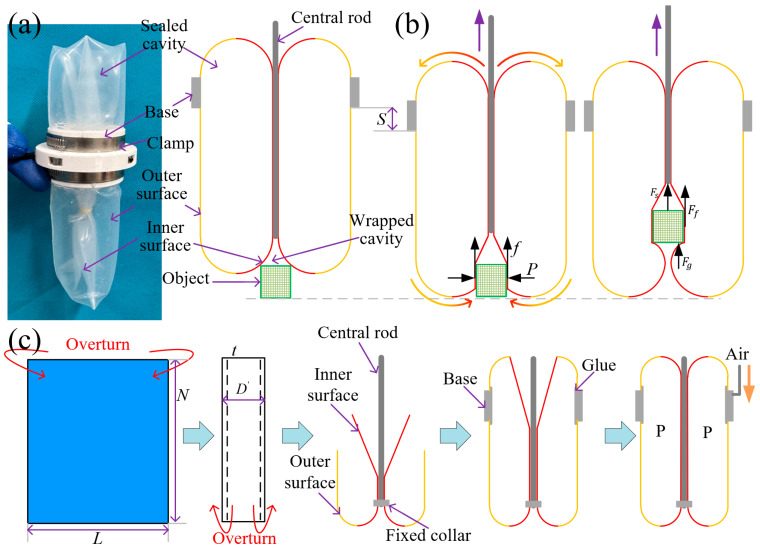
Prototype and fabrication process of the swallowing gripper. (**a**) Prototype and schematic diagram of the swallowing gripper. (**b**) Schematic diagram of the swallowing and grasping process. (**c**) Fabrication flow of the sealed cavity.

Figure 1a shows the structural schematic of the swallowing-type soft gripper’s principle prototype. The gripper is formed by rolling and folding a rectangular silicone elastic membrane, and its shape can be regarded as consisting of a hollow cylinder and two semicircular rings. The silicone elastic membrane is divided into an inner membrane (red line) and an outer membrane (yellow line). The inner membrane is closely attached to the central rod, and driven by the central rod, the inner membrane and the outer membrane can flip and transform into each other. The outer membrane is connected to the base, which provides support points for the mutual flipping. For instance, in Figure 1b, when the central rod moves upward, the inner membrane above the base will become the outer membrane, and the outer membrane below the base will become the inner membrane. When an object comes into contact with the elastic membrane, it will be swallowed into the gripper driven by frictional force, and the overall downward movement of the gripper is required to assist swallowing. When the central rod moves downward, the movement of the elastic membrane is exactly the opposite, and the object will be expelled from the interior of the gripper driven by the frictional force.

The fabrication process of the swallowing gripper prototype is illustrated in Figure 1c. First, a rectangular silicone elastic membrane with a length *L*, width *N*, and thickness *t* is rolled left and right to form a cylindrical shape with a diameter of $D^{\prime }$ and height of *N*. The overlapping area (with an overlap width of δ) is bonded using adhesive. Then, the cylindrical membrane is rolled up and down to form the inner and outer membranes, and the central rod is bound to the inner membrane with a rubber band. Finally, the inner and outer membranes are bonded to the base using adhesive, thereby forming a sealed chamber. The chamber is filled with fluid to inflate it. Thus, the fabrication of the principle prototype of the swallowing-type soft gripper is completed.

### 2.2. Analysis and Modeling of the Elastic Membrane

Since the silicone elastic membrane constituting the sealed cavity of the swallowing gripper is a hyperelastic material, it undergoes changes in volume and shape after fluid injection and object swallowing. Therefore, it is essential to analyze its volume and shape.

**Figure 2:**
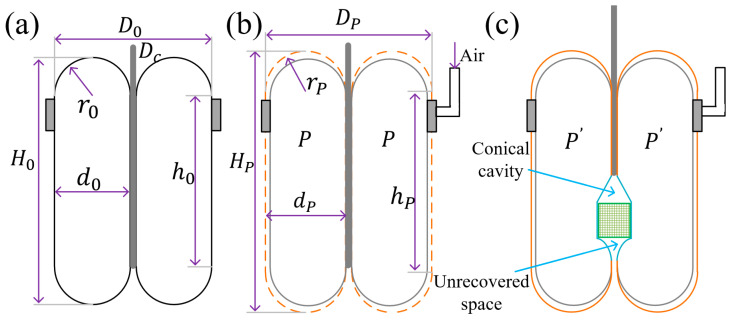
Schematic diagram of the deformation of the elastic membrane in the sealed cavity. Prototype and fabrication process of the swallowing gripper. (**a**) Original configuration. (**b**) Configuration after injecting the fluid medium (air). (**c**) Configuration after swallowing the object.

Assuming that the sealed cavity expands into an axisymmetric ideal shape after fluid injection, it can be regarded as consisting of a hollow cylinder and two semicircular rings, as illustrated in Figure 2. Before fluid injection, the sealed cavity has not yet expanded, and the following relational expressions hold:

(1) $$N=2h_{0}+2\pi r_{o}-L_{b}$$

(2) $$L=\pi D_{0}+\delta$$

(3) $$r_{0}=\frac{D_{0}-D_{c}}{4}$$

(4) $$d_{0}=2r_{0}$$

where $h_{0}$ denotes the height of the hollow cylinder, $r_{0}$ denotes the minor radius of the semicircular rings, $L_{b}$ denotes the width of the base, $D_{0}$ denotes the diameter of the gripper, and $H_{0}$ denotes the height of the gripper.

Therefore, the initial volume $V_{cylinder}$ of the hollow cylinder is given by:

(5) $$V_{cylinder}=\frac{\pi }{4}(D_{0}^{2}-D_{c}^{2})h_{0}$$

The initial volume $V_{ring}$ of the hollow cylinder is given by:

(6) $$V_{ring}=2\pi^{2}\frac{D_{0}-2r_{0}}{2}r_{0}^{2}$$

When the central rod drives the inner and outer membranes to flip, the volume of the central rod occupied in the sealed cavity undergoes a gradual change during its movement, and the rate of this change is affected by the moving speed of the central rod. Therefore, the volume $V_{rod}$ the central rod is given by:

(7) $$\begin{array}{cc} V_{rod}=\frac{\pi }{4}D_{c}^{2}\times h_{0}=\frac{\pi }{4}D_{c}^{2}\times v_{c}t, & 0\leq v_{c}t\leq h_{0} \end{array}$$

where $v_{c}$ denotes the moving velocity of the central rod. To ensure that the elastic membrane of the swallowing gripper can closely adhere to the object, while the central rod moves by a distance of $∆L_{c}$, the entire gripper also needs to move by a corresponding distance of S.

(8) $$\Delta L_{c}=2S$$

The ideal volume $V_{0}$ of the sealed cavity of the swallowing gripper in the initial state is given by:

(9) $$V_{0}=\frac{\pi }{4}D_{0}^{2}(h_{0}+v_{c}t)-\frac{\pi }{4}D_{c}^{2}h_{0}+\pi^{2}(D_{0}-2r_{0})r_{0}^{2}$$

As can be seen from Equation (9), with the movement of the central rod, the initial volume of the sealed cavity undergoes continuous changes. When the central rod moves upward, it induces the swallowing action of the gripper; when the central rod moves downward, it induces the expelling action of the gripper.

Since the sealed cavity is fabricated from a flexible silicone rubber elastic membrane, it is prone to deformation when subjected to internal pressure or external compression, thereby affecting the volume and shape of the elastic membrane. Therefore, investigating the shape change in the membrane is of great significance for studying the pressure variation in the sealed cavity during the gripper’s swallowing process. When fluid with a specified pressure is injected into the sealed cavity, the elastic membrane will undergo a certain amount of expansion, increasing the volume of the sealed cavity until it eventually reaches a stable state. For the convenience of establishing a numerical model, the following assumptions are made regarding the shape of the swallowing gripper: (1) The sealed cavity has an ideal regular and symmetric structure, which can be regarded as consisting of a hollow cylinder and two semicircular rings; (2) The elastic membrane is isotropic and expands/contracts isometrically under pressure; (3) The inner wall of the hollow cylinder does not undergo expansion due to the presence of the central rod.

**Figure 3:**
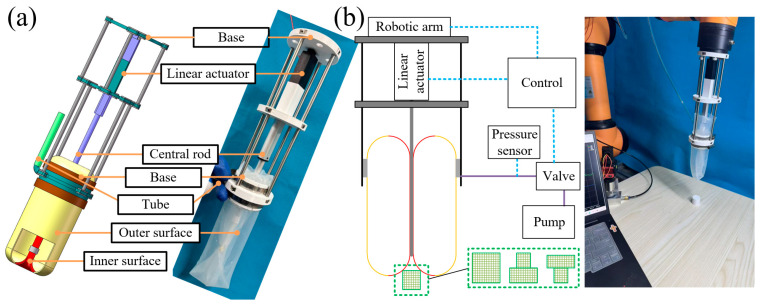
Sensing performance test. (**a**) swallowing gripper structure. (**b**) Sensing performance test platform.

When air at pressure P is injected into the sealed cavity, the elastic membrane will undergo axisymmetric expansion, as illustrated in Figure 3b. For the hollow cylinder, it experiences axisymmetric expansion under the action of internal pressure P, where $\lambda_{\theta }=r_{p}/r_{0}$ denotes the circumferential stretch ratio, $\lambda_{z}=H_{p}/H_{0}$ the axial stretch ratio, and $\lambda_{r}=t_{p}/t_{0}$ the radial stretch ratio. $r_{P}$, $H_{P}$, $t_{P}$ are the dimensional parameters of the elastic membrane after expansion, respectively. According to the incompressibility condition $\lambda_{\theta }\lambda_{z}\lambda_{r}=1\Rightarrow \lambda_{r}=1/\lambda_{\theta }\lambda_{z}$. Therefore, the deformed volume $V_{cylinder}^{P}$ of the cylinder in the sealed cavity is given by:

(10) $$V_{cylinder}^{p}=\pi r_{p}^{2}h_{p}=\pi {\left(r\lambda_{\theta }\right)}^{2}(h_{0}\lambda_{z})=V_{cylinder}\lambda_{\theta }^{2}\lambda_{z}$$

Due to the hyperelastic properties of the elastic membrane, Hooke’s Law fails to fully describe the membrane’s shape changes, while the Ogden model exhibits superior predictive ability in handling large-deformation materials such as rubber. The strain energy function of the Ogden model is given by:

(11) $$W=\sum_{i=1}^{N}\frac{\mu_{i}}{\alpha_{i}}(\lambda_{\theta }^{\alpha_{i}}+\lambda_{z}^{\alpha_{i}}+\lambda_{r}^{\alpha_{i}}-3)$$

By substituting the incompressibility condition $\lambda_{r}=1/\lambda_{\theta }\lambda_{z}$, it can be simplified to:

(12) $$W=\sum_{i=1}^{N}\frac{\mu_{i}}{\alpha_{i}}(\lambda_{\theta }^{\alpha_{i}}+\lambda_{z}^{\alpha_{i}}+{\left(\lambda_{\theta }\lambda_{z}\right)}^{-\alpha_{i}}-3)$$

Cauchy stress (Cauchy) can be given by:

(13) $$\begin{array}{cc} \sigma_{i}=\lambda_{i}\frac{\partial W}{\partial \lambda_{i}} & \left(i=\theta ,z,r\right) \end{array}$$

By substituting the principal stresses of the Ogden model into the circumferential stress equilibrium equation and the axial stress equilibrium equation, respectively, we have:

(14) $$\sigma_{\theta }=\sum_{i=1}^{N}\mu_{i}(\lambda_{\theta }^{\alpha_{i}}-{\left(\lambda_{\theta }\lambda_{z}\right)}^{-\alpha_{i}})-i=\frac{Pr_{0}\lambda_{\theta }^{2}\lambda_{z}}{t_{0}}$$

(15) $$\sigma_{z}=\sum_{i=1}^{N}\mu_{i}(\lambda_{z}^{\alpha_{i}}-{\left(\lambda_{\theta }\lambda_{z}\right)}^{-\alpha_{i}})-i=\frac{Pr_{0}\lambda_{\theta }^{2}\lambda_{z}}{2t_{0}}$$

By combining and simplifying Equations (10)–(15), the pressure-volume relationship of the hollow cylinder is obtained as:

(16) $$P{\left(V\right)}_{cylinder}=\frac{t}{r}\cdot \frac{1}{V}\sum_{i=1}^{N}\mu_{i}\left[{\left(\frac{V_{cylinder}^{P}}{V_{cylinder}}\right)}^{\alpha_{i}/2}-{\left(\frac{V_{cylinder}^{P}}{V_{cylinder}}\right)}^{-\alpha_{i}/2}\right]$$

Similarly, according to the Ogden model, the pressure-volume relationship of the semicircular ring is obtained as:

(17) $$P{\left(V\right)}_{ring}=\frac{t}{rR}\cdot \frac{1}{V_{ring}^{p}}\sum_{i=1}^{N}\mu_{i}\left[{\left(\frac{V_{ring}^{p}}{V_{ring}}\right)}^{\alpha_{i}/3}-{\left(\frac{V_{ring}^{p}}{V_{ring}}\right)}^{-\alpha_{i}/3}\right]$$

### 2.3. Establishment of the Swallowing Sensing Model

Due to the deformable characteristics of the elastic membrane of the swallowing gripper, collision and contact with external objects will alter the shape and volume of the gripper’s sealed cavity, thereby generating measurable fluid pressure differences. As the gripper grasps objects of different shapes, the trends of the pressure sensing curve during the swallowing process will also differ. We focus on discussing the pressure change responses generated by the swallowing gripper when swallowing cylindrical objects of different sizes, serving as an example to demonstrate the gripper’s sensing capability.

Assume that the diameter of the cylindrical object to be grasped is $D_{object}$ and its height is $H_{object}$. When the swallowing gripper swallows the object, a conical vacuum cavity will be formed at the top of the object; after the object is fully swallowed, an unrecovered space will be left below the object.

The swallowed volume of the object, denoted as $V_{object}$, varies with time:

(18) $$V_{object}=\frac{1}{4}\pi D^{2}h_{object}$$

where $h_{object}$ denotes the swallowed height of the object, which is related to the moving velocity of the central rod, $h_{object}=v_{c}t_{0}$, $t_{0}$ represents the time when swallowing starts after the gripper contacts the object. As the central rod moves, the gripper descends synchronously, and the frictional slip between the object and the elastic membrane can be neglected.

The volume of the conical cavity at the top of the object, denoted as $V_{con}$, is given by:

(19) $$V_{con}=\frac{1}{12}\pi D^{2}h_{con}$$

where $h_{con}$ denotes the height of the conical cavity.

Due to the intrinsic elasticity of the elastic membrane, an unrecovered space will be formed below the object, which is similar in shape to a cone. Its volume, denoted as $V_{unc}$, is given by:

(20) $$V_{unc}=\int_{r-\sqrt{A}}^{r}\pi \left(r-\sqrt{r^{2}-h^{2}}\right)dh=\frac{5}{3}r^{3}-2r^{2}\sqrt{A}$$

(21) $$A=rd_{0}-\frac{d_{0}^{2}}{4}$$

Therefore, during the gradual swallowing of the object, the volume change in the elastic membrane of the sealed cavity is given by:

(22) $$V=V_{P}-V_{object}-V_{con}-V_{unc}+V_{P}^{\prime }+V_{cen}$$

For a sealed cavity filled with an ideal gas, assuming a uniform distribution of pressure and temperature inside the cavity, and that the kinetic energy and potential energy of the air can be neglected, we obtain, according to Boyle’s Law:

(23) PV=k

where P denotes the pressure of the gas, V the volume of the gas, and k a constant.

By combining Equations (16), (17), (22) and (23), the pressure change formula generated by the sealed cavity swallowing the object is obtained as:

(24) $$P=\frac{P_{0}V_{0}}{V}-P{\left(V\right)}_{cylinder}-P{\left(V\right)}_{ring}$$

## 3. Experiments and Results

### 3.1. Sensing Performance Testing

To evaluate the sensing capability of the swallowing gripper, the proposed prototype was developed based on previous work [15], as shown in Figure 3a. A sensing performance test platform was established, as illustrated in Figure 3b. The swallowing gripper is connected to a robotic arm (AUBO-i5H, AUBO, Beijing, China) via a top base to control its overall movement, while the sealed cavity is sequentially connected to a pressure sensor (TFD-801, TEFUDE, Jinan, China, Range: −5 kPa~5 kPa, Accuracy: ±0.1%FS), a valve, and a pump through a tube. The linear actuator (EBH, Haijie, Xiancheng China, ST-802, 4 mm/s) of the gripper communicates with a controller to regulate the vertical movement of the central rod, thereby driving the inner and outer elastic membranes to invert and swallow the object. The pressure sensor real-time monitors the air pressure in the pipeline (i.e., the pressure inside the sealed cavity) and collaborates with the controller, the central rod, and the robotic arm to fulfill the operational requirements for object manipulation. In this performance test, cylindrical objects were used as the targets to be grasped, with diameters and heights of 15 mm, 20 mm, 25 mm, 30 mm, and 35 mm, respectively. Additionally, combined objects composed of two cylindrical objects of different sizes were supplemented as reference samples for comparison.

The test procedure for each object consists of several distinct phases: Movement stage: The robotic arm moves the swallowing gripper downward as a whole (speed: 2 mm/s) until it contacts the object. When the object is contacted, the volume of the sealed cavity undergoes an abrupt change, triggering a variation in the pressure sensor signal. Swallowing stage: The controller regulates the coordinated movement of the central rod and the robotic arm (upward speed of the central rod: 4 mm/s; downward speed of the robotic arm: 2 mm/s) to swallow and grasp the object until it is fully enclosed. Separation stage: When the object needs to be repositioned, the central rod moves in the reverse direction to actively release the object. Between the swallowing stage and the separation stage, the robotic arm can manipulate the swallowing gripper to perform movement, which is defined as the stable stage. Three repeated tests were performed for each object, and the test equipment and gripper were fully reset before each trial to minimize experimental errors.

**Figure 4:**
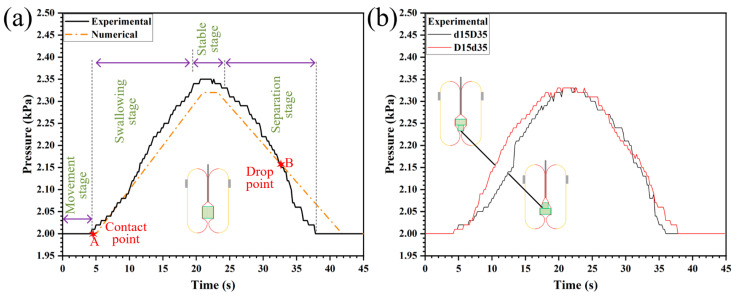
Sensing curve of single grasping. (**a**) Swallowing a cylinder. (**b**) Swallowing an assembly.

Figure 4 illustrates the pressure-time variation trend measured in a single test of the swallowing gripper swallowing an object. In Figure 4a, the red line represents the pressure change curve derived from the numerical model, while the black line denotes the measured pressure curve during the swallowing of a cylindrical object. During the movement stage, the elastic membrane of the gripper’s sealed cavity has not yet contacted the object, and the pressure inside the cavity remains constant at an initial value of 2 kPa. When the gripper contacts the object (Point A, contact point), the elastic membrane is forced to deform, causing a change in the volume of the sealed cavity and thus an increase in the internal air pressure. As the object is gradually swallowed, a conical space is formed by the elastic membrane above the object due to the pulling of the central rod. A cone-like rotational body is also formed below the object, as shown in Figure 2c. Precisely due to these two spaces, the pressure change curve described by the numerical simulation is relatively inconsistent with the curve measured in the actual experiment. During the separation stage, as the object is gradually released, the enclosed volume of the elastic membrane decreases progressively, leading to a gradual reduction in air pressure. At Point B, a sudden drop in pressure occurs, indicating that the object suddenly detaches from the enclosure cavity. This is because the frictional force generated by the air pressure inside the sealed cavity is insufficient to overcome the object’s own weight.

Figure 4b illustrates the pressure-time variation curves of the swallowing gripper swallowing a combined cylindrical object. The black line corresponds to the pressure curve when swallowing the convex-up combined object, while the red line represents the pressure curve when swallowing the convex-down combined object. From the two sets of experiments, it can be observed that although their pressure characteristics are consistent during the stable phase (due to the same enclosed volume), significant differences exist during the swallowing phase. The black line first increases slowly and then rises sharply, whereas the red line exhibits an initial sharp increase followed by a gradual rise.

**Figure 5:**
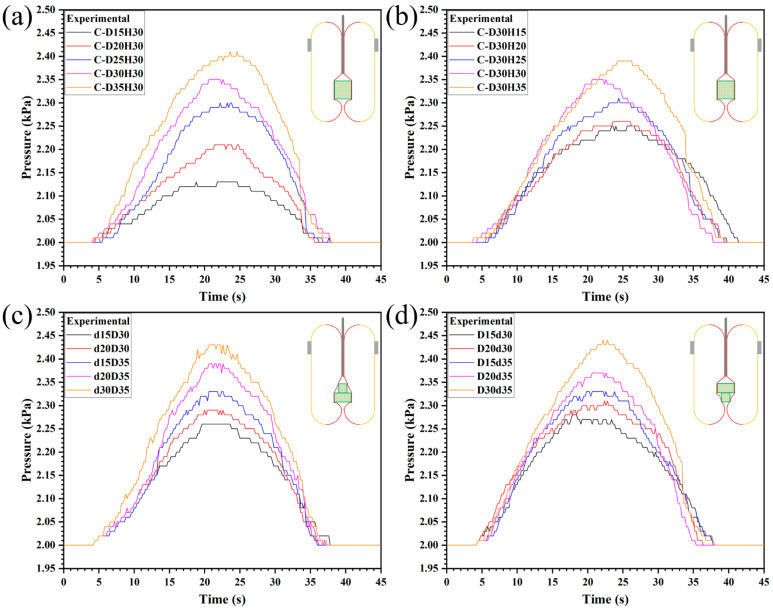
Sensitivity test. (**a**) Cylinders with different diameters (**b**) Cylinders with different heights. (**c**) Upright convex objects with different volumes. (**d**) Inverted convex objects with different volumes.

The sensitivity of the proposed sensing method was tested, focusing on the horizontal (diameter) and vertical (height) directions for objects with different shapes and volumes. Figure 5a presents the measured pressure feedback data when swallowing cylindrical objects with different diameters, with a diameter increment of 5 mm between consecutive samples. As observed from the figure, the curves are clearly distinguishable. Figure 5b illustrates the pressure feedback data for cylindrical objects with varying heights (height increment: 5 mm). Collectively, Figure 5a,b present the pressure feedback data for cylinders with different diameters and heights, respectively, both with a dimension increment of 5 mm. It can be observed that for the horizontal direction (diameter discrimination), the test data exhibit significant differences, indicating that the gripper can clearly perceive cylindrical objects with different diameters. In contrast, the test data show negligible differences for the vertical direction (height discrimination), suggesting that the gripper has lower sensitivity in the height direction. The distinct sensitivity between the two directions is attributed to the delayed recovery of the elastic membrane in the vertical direction during the object swallowing process. Figure 5c,d display the data obtained when swallowing different combined cylindrical objects. It can be seen that the pressure differences during the swallowing phase are insignificant; however, due to the varying volumes of the combined objects, the differences in the stable phase are relatively distinct.

**Figure 6:**
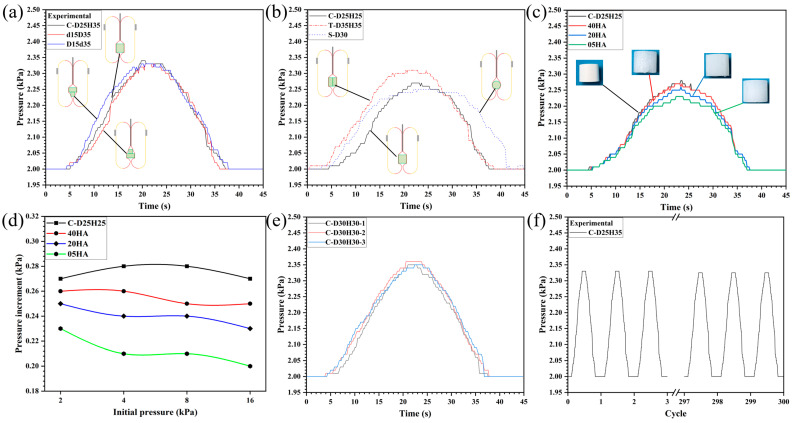
Performance test. (**a**) Cylinders, upright convex objects, and inverted convex objects with similar volumes. (**b**) Cylinders, triangular prisms, and spheres with similar volumes. (**c**) Cylinders of different hardness. (**d**) Different initial pressures. (**e**) Repeatability test. (**f**) Stability test.

Figure 6a shows the air pressure change curves of the sealed chamber during the swallowing of a cylinder, a triangular prism, and a sphere. For objects of different shapes, the characteristic pressure differences in their sealed chambers are significant, with an average pressure difference of 0.1 kPa, indicating that this method can achieve precise recognition of objects of various shapes. However, when the detected objects have similar shapes, sizes, and volumes, the pressure sensing method relying solely on the intrinsic relationship between volume and pressure has its limitations. Nevertheless, by detecting the internal air pressure of the sealed chamber in real-time during the object swallowing process, it is possible to differentiate objects of similar volumes based on the pressure change curves at different swallowing stages. Figure 6b also presents the swallowing perception curves of three objects with similar volumes (cylinder: 17,180.6 mm^3^, convex body: 17,082.4 mm^3^). According to these curves, the pressure values during the stable pressure phase for all three objects are consistent, but there are differences in the instantaneous pressure changes during the swallowing stage. Specifically, for the inverted convex body, since its larger end enters the swallowing channel first, the rate of volume change in the sealed chamber initially increases and then decreases; for the correctly oriented convex body, its smaller end completes swallowing first, so the rate of volume change in the sealed chamber shows an opposite trend, decreasing first and then increasing.

During the object swallowing process, the air pressure inside the sealed chamber continuously acts on the object, using friction to facilitate the object’s suction into the encapsulating chamber. Therefore, objects of different hardness will undergo compressive deformation during the swallowing process, resulting in a reduction in volume. Figure 6c shows the air pressure change curves of cylinders made from silicone materials with different Shore hardness values (40 HA, 20 HA, 5 HA) during the swallowing process. Softer objects (20 HA, 55 HA) experience significant volume deformation due to air pressure, leading to smaller pressure changes compared to harder objects (40 HA). Additionally, experiments were conducted on sealed chambers with different initial pressures to investigate the effect of initial pressure on the pressure difference generated during object grasping. The results are shown in Figure 6d. The experimental results indicate that as the initial pressure increases, the compressive effect on the object also increases, leading to a greater pressure difference.

For the measurement repeatability of the sensing method, multiple tests were performed on the same type of object. The test results, as shown in Figure 6e, indicate that the multiple measurement results are consistent. For the stability of the sensing method under long-term operation, a cyclic test of up to 300 cycles was conducted, with each cycle lasting 45 s. After the long-term stability test, no significant performance degradation was observed in the pressure change curve of each cycle, as shown in Figure 6f.

### 3.2. Closed-Loop Control with Fluidic Sensing

Based on an in-depth understanding of the sensing capability achieved through the intrinsic volume-pressure relationship, the swallowing gripper can be endowed with high-level manipulation tasks, such as the classification or sorting of objects based on their shape and size.

**Figure 7:**
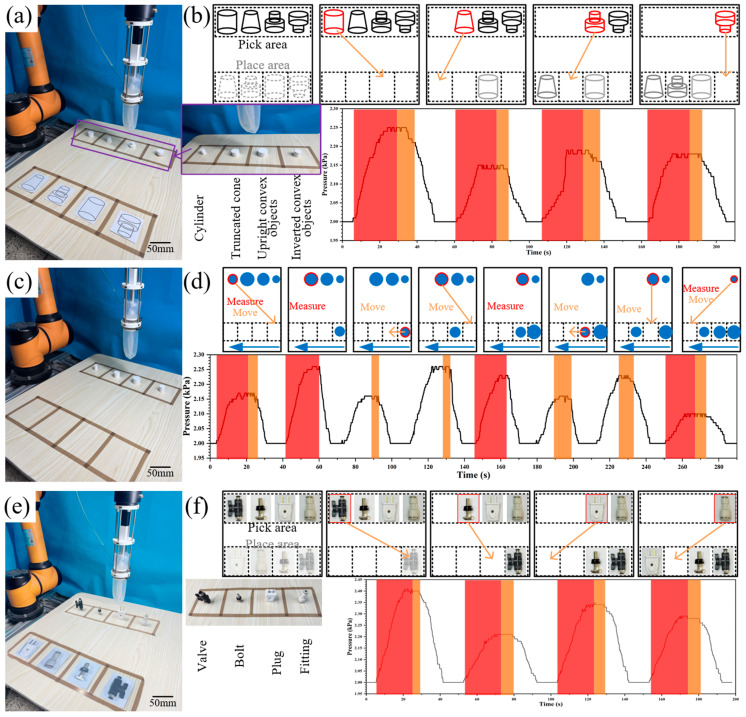
Intelligent grasping decision. (**a**) Classification grasping test platform. (**b**) Flow chart of the classification grasping test. (**c**) Size Sorting Test Platform. (**d**) Flow Chart of the Size Sorting Test. (**e**) Physical object grasping test platform. (**f**) Physical object grasping test flowchart.

To achieve the automatic classification and sorting of objects with different geometric shapes, objects of various shapes can be categorized based on the pressure-sensing time-history curves generated by the swallowing gripper during object grasping. Figure 7a presents the test platform for the classification and grasping experiment as well as the corresponding time-history data. The four types of objects classified include a cylinder, a truncated cone, a convex-up combined object, and a convex-down combined object, with a uniform total height of 20 mm for all samples. The cylinder has a diameter of 25 mm; the truncated cone features a base diameter of 25 mm and a top diameter of 15 mm; the convex-up/down combined objects consist of two cylindrical segments with diameters of 15 mm and 25 mm, respectively, and equal heights. These objects are 3D-printed using PLA material and randomly placed in the pick area. Object-specific symbols are marked in the place area, and their coordinates are pre-programmed into the algorithm. By controlling the robotic arm to drive the swallowing gripper for object grasping, the object’s shape is determined by comparing the measured pressure time-history curve during the swallowing process with the pre-stored pressure profiles in the algorithm. Subsequently, the gripper places the object in the corresponding designated region.

Figure 7b illustrates the pressure-time variation relationship during object grasping by the swallowing gripper in the object classification process. Although the two convex-shaped objects have the same volume during the experiment, slight differences exist in the pressure change curves generated during swallowing. For the convex-up combined object, the pressure curve first increases slowly after contact and then rises sharply, whereas the convex-down combined object exhibits the opposite trend. After 10 classification and placement tests, the correct placement success rate was 100% for both the cylinder and the truncated cone, 90% for the convex-up combined object, and 80% for the convex-down combined object. The reason for the misplacement of the convex-shaped objects is that they have the same volume, and classification relies solely on the pressure changes during the swallowing phase without pressure differences in the stable phase, leading to misjudgment.

Subsequently, we verified the sensing capability of the swallowing gripper using objects of the same shape but different diameters, aiming to test the gripper’s sensitivity in the horizontal direction, as shown in Figure 7c. Cylindrical objects with different diameters (15 cm, 20 cm, 25 cm, and 30 cm) were randomly placed in the pick area, with a uniform height of 20 cm. When swallowing a new object, its size is determined by measuring the feedback pressure in the gripper’s pipeline, and this pressure is averaged during the stable phase of the swallowing process. If the feedback pressure is greater than the pressures stored in the sorted array, the robotic arm rearranges the sorted queue: First, the object is placed down; subsequently, the already positioned objects are moved sequentially to create a new vacancy; finally, the object is repositioned in the formed vacancy. Otherwise, the robotic arm places the object directly at the end of the queue. This object size sorting strategy based on fluid pressure sensing is also applicable to objects of different shapes, enabling sorting by their volumes. Notably, the proposed sensing method does not require additional calibration procedures, only requiring a comparison of the pressure sensing differences between two swallowing processes for sorting.

Subsequently, practical grasping experiments were conducted using valves, bolts, plugs, and air tube connectors as test objects. Their volumes (measured by the displacement method) were 16 mL, 5 mL, 12 mL, and 8 mL, respectively, exhibiting significant volume differences. Figure 7f illustrates the pressure variation curve within the sealing chamber during the grasping and placement of these four objects by the swallowing gripper. Each grasping operation clearly revealed distinct volume and pressure differences. Across five repeated experiments, the gripper consistently and accurately completed grasping and precise placement, demonstrating excellent repeatability and adaptability.

### 3.3. Verification of Size Independence

**Figure 8:**
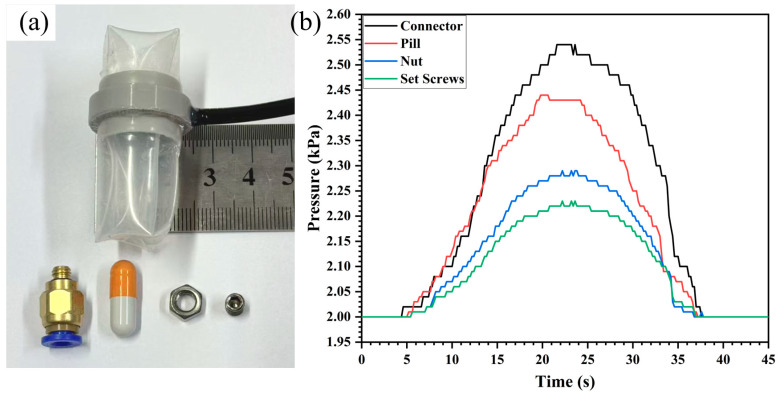
Verification of size independence. (**a**) Small-scale swallowing gripper prototype. (**b**) Small-scale pressure sensing curve.

This perception mechanism relies on pressure changes induced by the deformation of the gripper’s sealing chamber to detect object shapes. The gripper not only performs grasping functions but also utilizes pressure variations to achieve object perception. We further investigated whether the performance of the gripper during object swallowing at small scales remains consistent with that at large scales. An additional swallowing gripper with a diameter of 20 mm was fabricated. Due to the complexity of ancillary equipment, manual guidance of elastic film inversion was employed for the small-scale prototype to achieve swallowing, as illustrated in Figure 8a. A gas pressure of 2 kPa was injected into the sealing chamber, and various objects such as connectors, pills, nuts, and set screws were physically swallowed and grasped. The external tubing could detect pressure changes within the sealing chamber. The test results, shown in Figure 8b, indicate that the pressure change trends are consistent with those of the large-scale swallowing gripper, demonstrating that this method is not limited by size and is equally applicable to both small-scale and large-scale swallowing grippers. This finding confirms the size independence of the proposed mechanism, with test object volumes ranging from 20% to 80% of the sealing chamber volume exhibiting similar pressure response patterns.

## 4. Discussion

In summary, we propose a fluid pressure sensing strategy suitable for swallowing-wrapping type soft grippers. This strategy eliminates the need to embed sensing elements into the large-deformation and high-compliance elastic membrane, relying solely on the relationship between fluid pressure and volume deformation within the sealed cavity. Through a remotely connected pressure sensor, the interaction between the soft gripper and the object during the swallowing process can be accurately interpreted, thereby determining the object’s shape.

To achieve accurate fluid pressure sensing feedback, we established an expansion model for the deformation relationship of the elastic membrane in the gripper’s sealed cavity under initial pressure injection and during interaction with objects. Taking cylindrical objects as the targets to be grasped, a swallowing sensing model for the gripper was developed, and the volume-pressure variation relationship of the sealed cavity during the swallowing process was derived. During object swallowing, conical spaces are formed above and below the object. A fluid pressure sensing test platform was established to evaluate the sensing performance of the proposed swallowing gripper based on this sensing method, including sensitivity, resolution, repeatability, and stability during long-term operation. Additionally, the differences in pressure changes generated during swallowing for objects with the same volume but different shapes were tested. Finally, simple physical grasping demonstrations were conducted based on the proposed sensing method, including the classification of objects with different shapes and the sorting of objects with different sizes. The test results indicate that the gripper can determine the object’s shape based on the fluid pressure changes during the swallowing process.

This study only adopted air as the fluid medium of the sealed cavity. Future work will consider the pressure sensing model when using liquid as the fluid medium and simultaneously investigate its object sensing capability underwater.

## Data Availability

The original contributions presented in this study are included in the article. Further inquiries can be directed to the corresponding author.
